# Short-Term Outcomes of Coronary Endarterectomy as an Adjunct to Coronary Artery Bypass Grafting: A Systematic Review and Meta-Analysis of Over 100 000 Patients

**DOI:** 10.1093/icvts/ivag091

**Published:** 2026-03-25

**Authors:** Joshua J Hon, Arian Arjomandi Rad, Archie Egrilmezer, Fadi Ibrahim Al-Zubaidi, Andrea D’Alessio, Shivika Sharma, Mariam Omar, Vasiliki Androutsopoulou, Sadeq Ali-Hasan-Al-Saegh, Alexander Weymann, Arjang Ruhparwar, Peyman Sardari Nia, Thanos Athanasiou, Antonios Kourliouros

**Affiliations:** Department of Surgery and Cancer, Imperial College London, London, W12 0NN, United Kingdom; Department of Cardiothoracic Surgery, Oxford University Hospitals NHS Foundation Trust, Oxford, OX3 9DU, United Kingdom; Department of Cardiothoracic Surgery, Maastricht University Medical Center+, Maastricht, 6229 HX, The Netherlands; Department of Surgery and Cancer, Imperial College London, London, W12 0NN, United Kingdom; Department of Cardiothoracic Surgery, Oxford University Hospitals NHS Foundation Trust, Oxford, OX3 9DU, United Kingdom; Department of Cardiothoracic Surgery, Oxford University Hospitals NHS Foundation Trust, Oxford, OX3 9DU, United Kingdom; Department of Cardiothoracic Surgery, Oxford University Hospitals NHS Foundation Trust, Oxford, OX3 9DU, United Kingdom; Department of Cardiothoracic Surgery, Oxford University Hospitals NHS Foundation Trust, Oxford, OX3 9DU, United Kingdom; Department of Cardiothoracic Surgery, University General Hospital of Larissa, Larissa, 41110, Greece; Department of Cardiothoracic, Transplantation and Vascular Surgery, Hannover Medical School, Hannover, 30625, Germany; Department of Cardiothoracic, Transplantation and Vascular Surgery, Hannover Medical School, Hannover, 30625, Germany; Department of Cardiothoracic, Transplantation and Vascular Surgery, Hannover Medical School, Hannover, 30625, Germany; Department of Cardiothoracic Surgery, Maastricht University Medical Center+, Maastricht, 6229 HX, The Netherlands; Department of Surgery and Cancer, Imperial College London, London, W12 0NN, United Kingdom; Department of Cardiothoracic Surgery, Oxford University Hospitals NHS Foundation Trust, Oxford, OX3 9DU, United Kingdom

**Keywords:** coronary endarterectomy, coronary artery bypass grafting, cardiac surgery, coronary artery disease

## Abstract

**Objectives:**

To assess short-term outcomes of coronary artery bypass grafting (CABG) with adjunct coronary endarterectomy (CE) compared with isolated bypass grafting, and to synthesize available confounder-adjusted effect estimates.

**Methods:**

We conducted a systematic review and meta-analysis following PRISMA guidelines. MEDLINE, Embase, and CENTRAL were searched from January 2000 to June 2025. Eligible studies compared adult patients undergoing CABG with CE versus isolated CABG. Two reviewers independently screened studies, extracted data, and assessed quality. Random-effects meta-analysis was performed. The primary outcome was 30-day or in-hospital mortality.

**Results:**

Sixteen studies (119 458 patients) were included. CABG with CE was associated with higher mortality (RR 1.84, 95% CI 1.65-2.04). Pooling adjusted odds ratios from 3 studies yielded OR 1.76 (95% CI 1.55-2.00), with 2 of 3 individual estimates not reaching significance. Secondary outcomes showed increased risks of perioperative myocardial infarction (RR 1.99, 95% CI 1.29-3.07), stroke (RR 1.37, 95% CI 1.08-1.75), renal failure (RR 1.62, 95% CI 1.44-1.82), and intra-aortic balloon pump use (RR 1.96, 95% CI 1.41-2.70). Sensitivity analyses confirmed consistency across all the subgroups.

**Conclusions:**

CABG with CE is associated with higher short-term mortality and morbidity compared with isolated bypass grafting; however, confounder-adjusted analyses suggest this excess risk is partly attributable to greater baseline disease severity rather than an independent procedural effect. The scarcity of data and absence of randomized evidence preclude definitive causal conclusions. These findings provide benchmarking data for counselling when endarterectomy is necessary to achieve complete revascularization.

## Graphical Abstract

**Figure:**
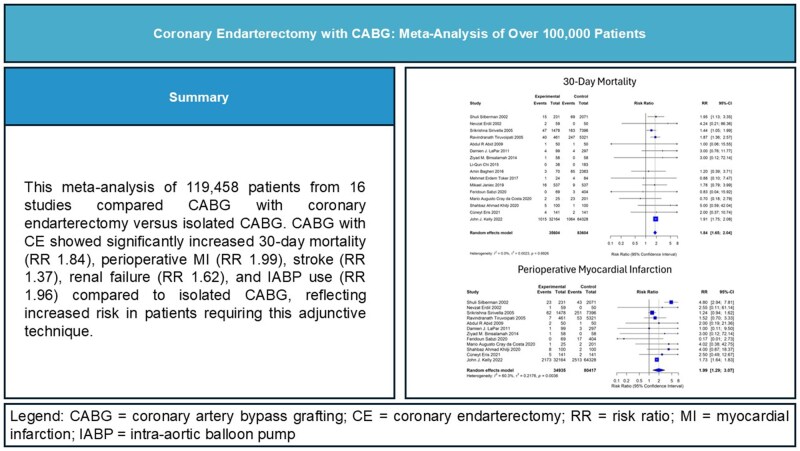

## INTRODUCTION

Coronary endarterectomy (CE) is an adjunctive surgical technique employed during coronary artery bypass grafting (CABG) to treat vessels with severe, diffuse atherosclerotic disease that are not amenable to standard bypass grafting techniques.^1^ The procedure involves removing atherosclerotic plaque from the coronary lumen, creating a smooth surface for graft anastomosis and potentially improving long-term patency.^2^

First described in the 1950s,^3^ the procedure gained popularity in subsequent decades but later experienced reduced utilization due to early concerns about increased perioperative morbidity and mortality, along with questions regarding long-term graft patency. Despite these historical concerns, CE remains an important technique for achieving complete revascularization in patients with complex coronary artery disease (CAD) who would otherwise be unsuitable for conventional bypass surgery.

The decision to perform CE is typically reserved for patients with extensive CAD characterized by long segments of diffuse stenosis, heavy calcification preventing adequate anastomosis, small vessel calibre with widespread disease, or previous failed percutaneous interventions with extensive in-stent restenosis. The most commonly treated vessels include the left anterior descending (LAD) artery and right coronary artery (RCA),^4^ though any vessel may be subject to endarterectomy when clinically indicated.

Despite decades of clinical use, the safety and effectiveness of CABG with CE remains incompletely understood. Recent evidence suggests surgeon proclivity for the procedure varies substantially, with patients operated on by frequent-endarterectomisers demonstrating marginally reduced survival compared with those operated on by occasional or never-endarterectomisers.^5^ This uncertainty stems from several factors, including the inherent complexity of patient selection whereby those requiring the procedure have more severe, diffuse coronary disease compared to those suitable for isolated CABG. Additionally, most existing studies are observational with small sample sizes and heterogeneous populations, whilst surgical techniques, perioperative care, and patient selection criteria have evolved over time.

Given the complexity of CAD requiring endarterectomy and the evolving evidence base, a comprehensive systematic review and meta-analysis is needed to synthesize the available evidence and assess the outcomes of CABG with CE using isolated CABG as a reference standard. This approach acknowledges the inherent differences between patient populations whilst providing clinically relevant benchmarks for understanding results associated with this adjunctive procedure. The primary objective of this systematic review was to assess short-term clinical outcomes in patients undergoing combined procedures, using outcomes from isolated CABG as a comparator to contextualize the safety profile and clinical effectiveness of this adjunctive technique.

## METHODS

### Protocol registration and reporting standards

This systematic review was conducted and reported according to the Preferred Reporting Items for Systematic Reviews and Meta-Analyses (PRISMA) guidelines.^6^ The protocol was registered with PROSPERO (International Prospective Register of Systematic Reviews) prior to commencing the systematic review with registration ID: CRD420251066909.

### Eligibility criteria

Studies were eligible for inclusion if they involved adult patients aged 18 years or older undergoing CABG. All included studies were required to provide direct comparisons between patients who underwent CABG with CE and those who received isolated CABG. No restrictions were placed on patient risk profiles, comorbidities, or coronary anatomy complexity to ensure comprehensive capture of the available evidence.

We included CABG combined with CE using any recognized technique. This included open endarterectomy with or without patch reconstruction, traction endarterectomy, gas endarterectomy, or combined techniques. Studies were included regardless of the number of vessels subject to endarterectomy or the type of graft used, allowing for broad representation of current clinical practice patterns.

Control patients received standard CABG without endarterectomy. Similar to the intervention group, no restrictions were placed on the number of bypass grafts performed or the type of graft conduits employed, ensuring that studies reflecting diverse surgical approaches could be included.

The primary end-point was 30-day or in-hospital mortality, whilst secondary outcomes encompassed a range of perioperative complications including myocardial infarction (MI), stroke, renal failure, and reoperation for bleeding. Additional secondary outcomes included length of stay measures, intra-aortic balloon pump (IABP) use, and postoperative atrial fibrillation.

Both randomized controlled trials and non-randomized comparative studies were eligible. All studies were required to provide direct comparisons between the CABG with CE and isolated CABG groups, with a minimum follow-up period of 30 days to capture short-term outcomes adequately.

### Exclusion criteria

Case reports and case series without comparison groups, single-arm studies without comparative control groups, studies focusing exclusively on paediatric populations, studies of emergency or salvage procedures without planned comparison, conference abstracts without full-text availability, studies where aforementioned primary and secondary outcomes could not be extracted, studies published before January 2000, and studies in languages other than English were excluded.

### Information sources and search strategy

A comprehensive literature search was conducted in MEDLINE (via PubMed), Embase, and the Cochrane Central Register of Controlled Trials (CENTRAL) from January 1, 2000 to June 2025. The search strategy was developed including core concepts of CABG, CE, and comparative study designs. Additional sources included reference lists of included studies and relevant review articles.

### Study selection and data extraction

Two independent reviewers (J.J.H. and A.E.) screened all titles and abstracts, followed by full-text review using standardized forms. Disagreements were resolved through discussion reaching a consensus. Data extraction was performed independently by 2 reviewers using a standardized form, capturing study characteristics, population demographics, intervention details, and outcome data.

### Quality assessment

Study quality was assessed using the Newcastle-Ottawa Scale (NOS) for non-randomized studies.^7^ Two independent reviewers assessed the quality of each included study, with disagreements resolved through discussion.

### Statistical analysis

Random-effects meta-analysis was performed using the DerSimonian-Laird method.^8^ Risk ratios with 95% confidence intervals (CIs) were calculated for dichotomous outcomes, and mean differences for continuous outcomes. Statistical heterogeneity was assessed using the *I*^2^ statistic and Chi^2^ test. Subgroup analyses were conducted by endarterectomy technique and patient age categories. Meta-regression was performed to explore the relationship between study-level characteristics and treatment effects. Publication bias was assessed using Egger’s test, funnel plots, and Doi plots with calculation of the Luis Furuya-Kanamori (LFK) index.^9^^,^^10^ We performed complete-case analysis for all outcomes, analysing only studies that reported each specific end-point. Studies with missing secondary outcome data were included in the review if they reported the primary outcome. All analyses were conducted in R version 4.5.0 with the “meta” and “metafor” packages.^11–13^

### Adjusted effect estimates

Where available, we extracted adjusted effect estimates from multivariable regression analyses or propensity score-matched comparisons. We prioritized adjusted estimates over crude comparisons when both were reported. Studies were considered as providing adjusted analysis if they reported effects from logistic regression, Cox proportional hazards models, or propensity score matching methods. We documented the covariates adjusted for in each analysis. Adjusted estimates were pooled separately from crude estimates using random-effects meta-analysis when at least 2 studies reported comparable adjusted effect measures for the same outcome.

### Pooled adjusted analysis

Studies reporting between-group adjusted odds ratios for operative mortality were pooled using a random-effects inverse-variance model (DerSimonian-Laird) to provide a confounder-adjusted estimate of the association between CE-CABG and early mortality. Heterogeneity among adjusted estimates was assessed with the *Q* statistic and *I*^2^ index.

### Sensitivity analyses

To assess the robustness of the primary result, sensitivity analyses were performed restricting to: (1) studies reporting long-term follow-up data; (2) studies reporting CE technique details; (3) studies reporting vessel distribution data; (4) studies with any form of adjusted analysis; (5) studies reporting between-group adjusted odds ratios; (6) studies explicitly confirming isolated CABG populations; and (7) all studies excluding Kelly 2022 (the largest contributing study).

## RESULTS

### Study selection and characteristics

**Figure 1.**
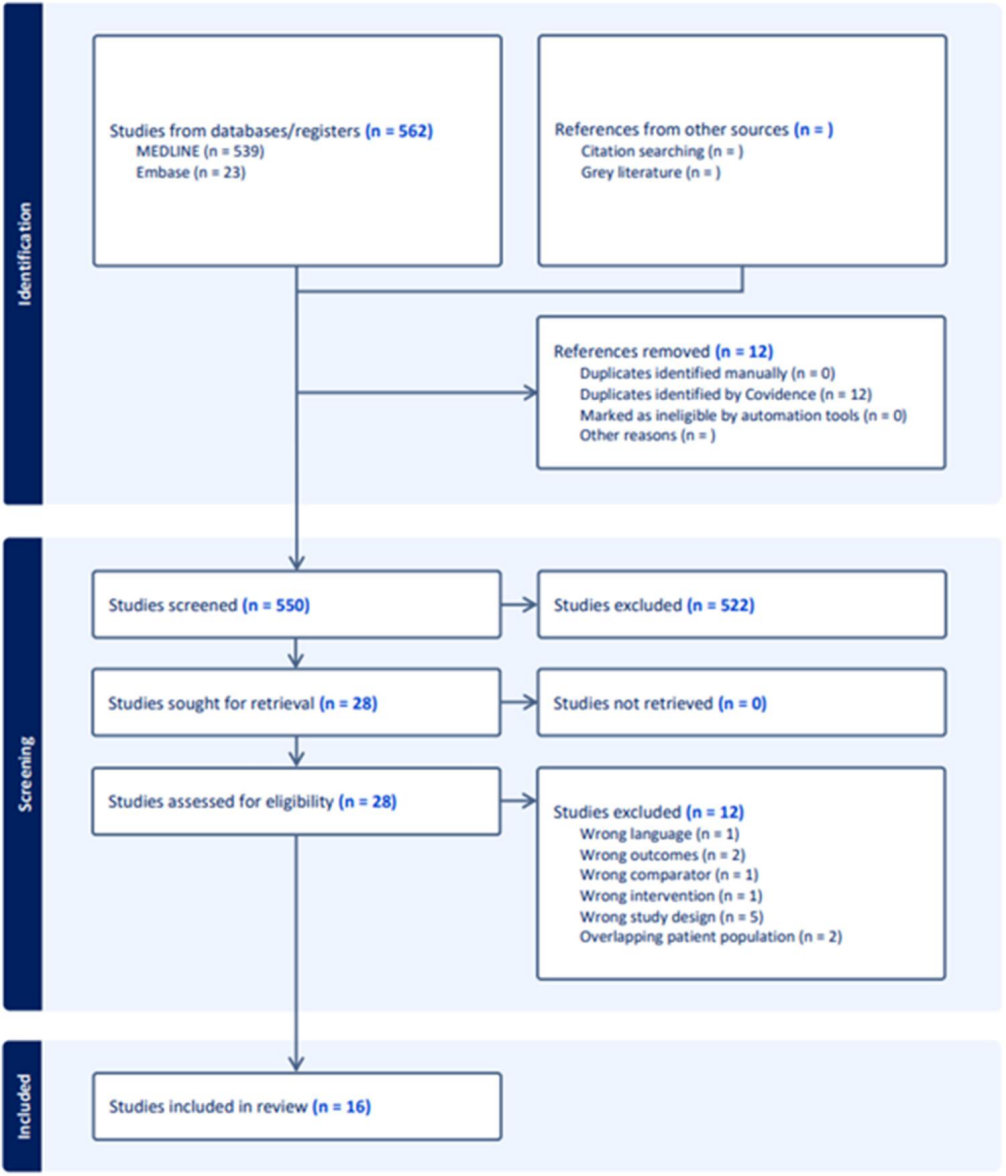
PRISMA Flow Diagram.

The systematic search identified 562 studies from databases, with 16 studies ultimately meeting inclusion criteria after screening and full-text review. The PRISMA flow diagram details the selection process (**Figure 1**), with 522 studies excluded during screening and 12 studies excluded during full-text assessment for various reasons including wrong study design, wrong outcomes, and overlapping patient populations.

### Quality assessment

The quality assessment of the 16 included studies using the Newcastle-Ottawa Scale revealed predominantly fair to good quality evidence. The majority of studies (*n* = 12, 70.6%) achieved fair quality ratings, while 4 studies (23.5%) were rated as good quality, and only 1 study (5.9%) was classified as poor quality (**Table S1**).

### Study characteristics

The 16 included studies comprised primarily retrospective cohort studies conducted between 2000 and 2025 (**Table 1**).^4^^,^^14–29^ One study (Kelly 2022) was notably larger than others, representing a Society of Thoracic Surgeons database analysis that contributed substantial weight to the overall analysis. Given the potential influence of this large study on overall results, analyses were conducted both including and excluding this study to assess consistency of findings.

**Table 1.** Study Characteristics

Study	Country	*N*	***N* (CE** **+** **CABG)**	Age	Male (%)	LVEF (%)	Diabetes mellitus (%)	Previous MI (%)	Hypertension (%)	CKD (%)	Peripheral vascular disease (%)
Kelly 2022	United States, Canada	96 427	32 164	65.0 ± 10.1	76.1	51.6 ± 12.3	52.6	53.5	NR	3.4	14.8
Eris 2021	Turkey	282	141	64.5 (weighted median)	68.8	55 (weighted median)	22	NR	41.8	NR	16.3
Sabzi 2020	Iran	473	69	58.3 ± 9.6	74.2	44.4 ± 11.1	26.8	9.9	45.5	NR	NR
CostaMACD 2020	Brazil	226	25	NR	74.3	NR	35	NR	NR	3.1	NR
Janiec 2019	Sweden	1074	537	66.1 ± 8.8	79.4	NR	NR	NR	NR	NR	NR
Bagheri 2016	Iran	2452	70	58.9 ± 10.0	72.4	46.9 ± 14.3	35	35.4	50.3	3.1	NR
Chi 2015	China	221	38	68.9 ± 6.4	62.4	46.7 ± 11.9	43	NR	66.2	NR	49.3
Binsalamah 2014	Canada	116	58	66.9 ± 9.9	90	46.3 ± 9.6	NR	NR	74	4.3	10.5
LaPar 2011	United States	586	99	62.05 ± 11.1	71.5	NR	43.1	54.8	NR	5.3	16.2
Abid 2009	Pakistan	100	50	54.79 ± 7.8	97	46.3 ± 11.5	36	NR	43	NR	NR
Sirivella 2005	United States	6874	1478	65.3 ± 5.2	71.2	48 ± 14	34.8	62.3	73.7	5.7	23.7
Tiruvoipati 2005	United States	5782	461	63.46 ± 9.21	80.4	NR	17.5	51.7	45.1	45.5	13.7
Silberman 2002	Israel	2302	231	63.8 ± 10.0	77.9	NR	35.7	62.3	53.3	7	16.8
Erdil 2002	Turkey	109	59	59.5 (median)	88.1	NR	19.3	67	25.7	NR	NR
Khilji 2020	Pakistan	200	100	56.83 ± 3.85	72.5	42.93 ± 7.08	62.5	NR	37.5	NR	NR
Toker 2017	Turkey	108	24	63.31 ± 9.47	69.4	NR	45.4	NR	NR	5.6	NR

Missing data varied across studies and outcomes. All 16 studies reported early mortality (primary outcome). For secondary outcomes, reporting completeness ranged from 87.5% of studies for postoperative MI to 50% for anticoagulation protocols. Long-term mortality was reported by 10 studies (62.5%), whilst procedural details such as endarterectomy technique and patch reconstruction were documented in 8 studies (50%) and 7 studies (43.8%) respectively.

Across 6 studies reporting detailed vessel distributions (2961 endarterectomized vessels), the LAD artery (36.7%) and RCA (34.6%) were most frequently treated, followed by circumflex and obtuse marginal branches (13.5%). The majority of patients (63%) underwent single-vessel endarterectomy, whilst 37% required multivessel endarterectomy, including 12% with 3-vessel procedures.

Surgical technique varied across studies. Among 5 studies clearly reporting technique (2463 vessels), traction or closed endarterectomy was employed in 79.9% of cases, whilst open endarterectomy with extended arteriotomy was used in 20.1%. Patch reconstruction was used selectively, primarily for extended arteriotomies, with either saphenous vein or internal thoracic artery patches employed.

### Primary outcome: 30-day/in-hospital mortality

**Figure 2.**
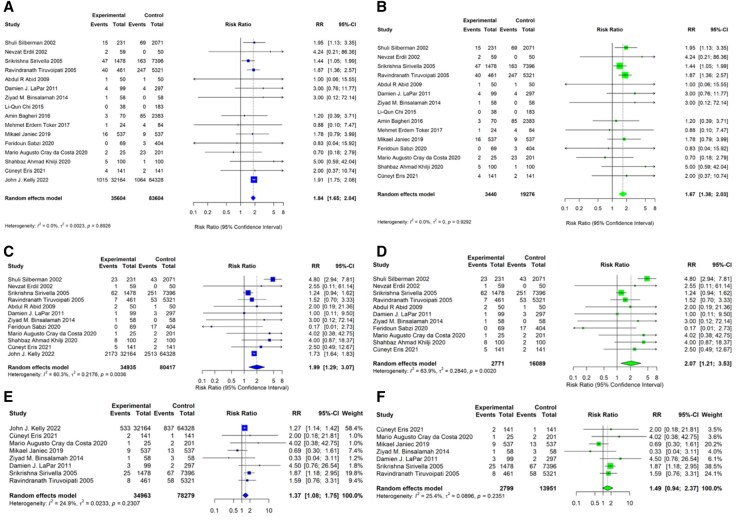
Forest Plots for the Primary Outcome of 30-day or in-Hospital Mortality and Key Secondary Outcomes. (A) 30-day or in-hospital mortality including Kelly 2022. (B) 30-day or in-hospital mortality excluding Kelly 2022. (C) Perioperative myocardial infarction including Kelly 2022. (D) Perioperative myocardial infarction excluding Kelly 2022. (E) Perioperative stroke including Kelly 2022. (F) Perioperative stroke excluding Kelly 2022

The analysis of 30-day or in-hospital mortality included all 16 studies. When including the Kelly 2022 study, the pooled analysis demonstrated a significantly increased risk of mortality with CABG plus CE compared to isolated CABG (RR 1.84, 95% CI 1.65-2.04). The analysis excluding Kelly 2022 showed a similar direction of effect but with a somewhat attenuated risk ratio (RR 1.67, 95% CI 1.38-2.03). Statistical heterogeneity was low in both analyses, with no significant heterogeneity detected (**Figure 2**).

### Adjusted effect estimates

Six studies reported adjusted effect estimates from multivariable regression or propensity score-matched analyses. Three of these provided between-group adjusted odds ratios for operative mortality and are pooled in the next subsection. Of the remaining individual-level findings, Kelly et al demonstrated that long-term mortality risk was time-dependent, with increased hazard only in the first year (HR 1.46, 95% CI 1.29-1.65) but not thereafter (1-3 years: HR 0.99, 95% CI 0.84-1.17; >3 years: HR 0.86, 95% CI 0.69-1.08). Similarly, the hazard ratio for MI was elevated in the first 3 years (HR 1.49 and 1.36) but not beyond (HR 1.03, 95% CI 0.58-1.81).^4^

Two studies reported predictors of adverse outcomes within endarterectomy cohorts only. Sirivella et al identified age ≥70 years (OR 1.04, *P* < .0001), ejection fraction <0.30 (OR 1.8, *P* < .0001), cardiopulmonary bypass time (OR 1.6, *P* = .002), and end-stage renal disease (OR 4.94, *P* < .0001) as independent mortality predictors.^24^ Silberman et al similarly found low cardiac output (OR 4.49, *P* < .0001), perioperative MI (OR 10.91, *P* < .0001), and renal failure (OR 5.54, *P* < .0001) were strong predictors, whilst endarterectomy of the LAD artery specifically increased perioperative infarction risk (OR 3.45, *P* = .014).^26^

### Pooled adjusted analysis for operative mortality

**Figure 3.**
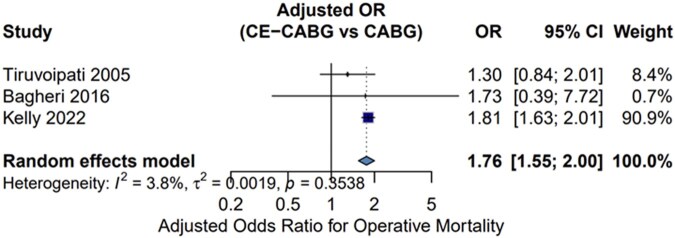
Forest Plot of Pooled Adjusted Odds Ratios for Operative Mortality from Three Studies Reporting Between-Group Confounder-Adjusted Estimates.

Three studies provided between-group adjusted odds ratios for 30-day or in-hospital mortality. Tiruvoipati et al reported an adjusted OR of 1.30 (95% CI 0.84-2.01) from multivariable logistic regression^25^; Bagheri et al reported an adjusted OR of 1.73 (95% CI 0.39-7.72) from stepwise multivariable regression^19^; and Kelly et al reported an adjusted OR of 1.81 (95% CI 1.63-2.01) from propensity score-matched logistic regression with generalized estimating equations, accounting for hospital-level clustering.^4^ The pooled random-effects adjusted OR was 1.76 (95% CI 1.55-2.00; **Figure 3**). Notably, 2 of the 3 individual adjusted estimates did not reach statistical significance, and the pooled adjusted OR (1.76) was numerically lower than the crude pooled RR (1.84), suggesting that part of the observed excess mortality risk is attributable to confounding by baseline disease severity rather than to the CE procedure itself.

### Sensitivity analyses

The direction and magnitude of the primary result were consistent across all sensitivity analyses (**Table 2**). Restricting to the 14 studies explicitly confirming isolated CABG populations yielded an RR of 1.87 (95% CI 1.73-2.03). Excluding Kelly 2022 attenuated the pooled RR to 1.67 (95% CI 1.38-2.03). Restricting to the 11 studies reporting CE technique yielded an RR of 1.54 (95% CI 1.20-1.98). Restricting to the 6 studies with vessel distribution data yielded an RR of 1.87 (95% CI 1.73-2.03). All other subgroup restrictions produced estimates within the range of 1.54-1.90, with *I*^2^ consistently at 0%, confirming the consistency of the primary findings.

**Table 2.** Sensitivity Analyses for Operative Mortality (CE-CABG Versus Isolated CABG)

Analysis	*N*	Effect estimate (95% CI)	*I*² (%)
Primary analysis (all studies)	16	RR 1.84 (1.65-2.04)	0.0
Excluding Kelly 2022	15	RR 1.67 (1.38-2.03)	0.0
Long-term follow-up reported	9	RR 1.87 (1.73-2.03)	0.0
CE technique reported	11	RR 1.54 (1.20-1.98)	0.0
Vessel distribution reported	6	RR 1.87 (1.73-2.03)	0.0
Any adjusted analysis available	7	RR 1.87 (1.73-2.02)	0.0
Between-group adjusted OR available (crude RR)	3	RR 1.90 (1.75-2.06)	0.0
Explicitly isolated CABG	14	RR 1.87 (1.73-2.03)	0.0
Pooled adjusted OR	3	OR 1.76 (1.55-2.00)	3.8

Abbreviation: *N*, number of studies contributing to the analysis.

Reporting completeness was also assessed, with findings summarized in **Table S2**.

### Secondary outcomes

#### Perioperative myocardial infarction

Analysis of perioperative MI included 14 studies. Both analyses demonstrated a significant increase in perioperative MI risk with CABG plus CE compared to isolated CABG. The pooled analysis including Kelly 2022 showed increased risk (RR 1.99, 95% CI 1.29-3.07), whilst the analysis excluding Kelly 2022 demonstrated a similar significant increase in perioperative MI risk (RR 2.07, 95% CI 1.21-3.53).

#### Perioperative stroke

Analysis of stroke outcomes included 9 studies. The analysis including Kelly 2022 showed a significant increase in stroke risk associated with CABG plus CE (RR 1.37, 95% CI 1.08-1.75), whilst the analysis excluding Kelly 2022 demonstrated no significant difference between the groups (RR 1.49, 95% CI 0.94-2.37).

#### Perioperative renal failure

The analysis of renal failure included 7 studies. Results showed increased risk with CABG plus CE in both analyses (including Kelly: RR 1.62, 95% CI 1.44-1.82; excluding Kelly: RR 1.43, 95% CI 1.12-1.82).

#### Reoperation for bleeding

Analysis included 5 studies. The analysis including Kelly 2022 showed a significant increase in reoperation risk associated with CABG plus CE (RR 1.32, 95% CI 1.02-1.70), whilst the analysis excluding Kelly 2022 demonstrated no significant difference between the groups (RR 1.05, 95% CI 0.69-1.59).

#### ICU length of stay

Analysis of intensive care unit (ICU) length of stay included 9 studies (Kelly 2022 did not report this outcome). The pooled analysis showed a near significant difference in ICU length of stay between groups (MD 0.61, 95% CI 0.21-1.01 days).

#### IABP use

Analysis of IABP use included 8 studies. Kelly 2022 did not report this outcome. The pooled analysis demonstrated a significant increase in IABP requirement associated with CABG plus CE compared to isolated CABG (RR 1.96, 95% CI 1.41-2.70).

#### Postoperative atrial fibrillation

Analysis of postoperative atrial fibrillation included 8 studies. Kelly 2022 did not report this outcome. The analysis showed no significant difference between the groups (RR 1.21, 95% CI 0.95-1.53).

**Figure 4.**
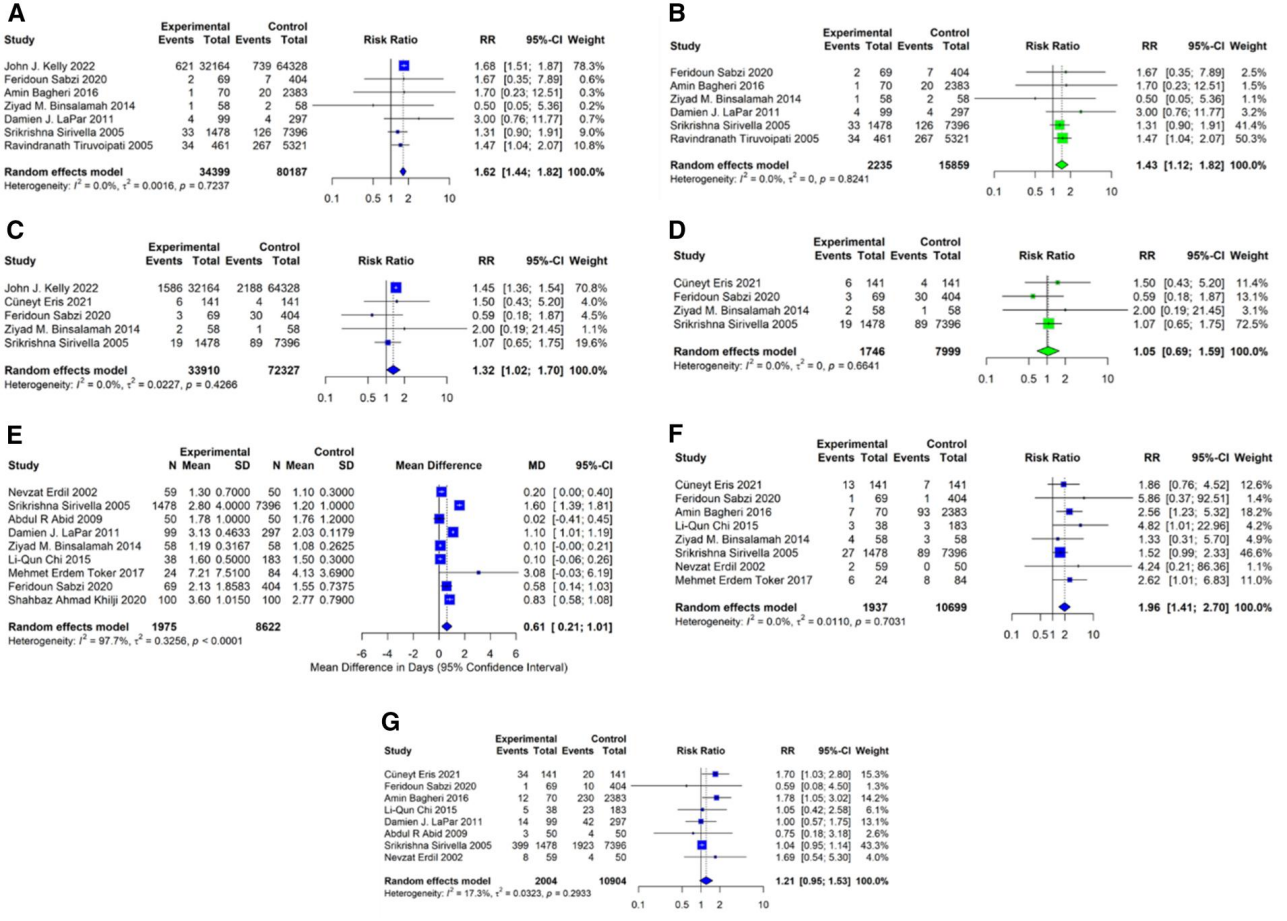
Forest Plots for Remaining Secondary Outcomes Comparing CABG with CE to Isolated CABG. (A) Perioperative renal failure including Kelly 2022. (B) Perioperative renal failure excluding Kelly 2022. (C) Reoperation for bleeding including Kelly 2022. (D) Reoperation for bleeding excluding Kelly 2022. (E) ICU length of stay. (F) Intra-aortic balloon pump use. (G) Postoperative atrial fibrillation

Secondary outcomes are summarized in **Figures 2** and **4**.

### Subgroup analyses and meta-regression

Subgroup analysis by endarterectomy technique revealed significant differences between open and traction endarterectomy techniques (*P* = .003), with open endarterectomy showing higher risk ratios (RR 1.56, 95% CI 1.20-2.01) compared to traction endarterectomy (RR 1.45, 95% CI 0.56-3.76). Age-based subgroup analysis demonstrated a paradoxically higher risk in younger patients (≤65 years: RR 1.90, 95% CI 1.75-2.06) compared to older patients (>65 years: RR 1.60, 95% CI 1.24-2.07; *P* < .001) (**Table 3**).

**Table 3.** Results of Subgroup Analyses for 30-day or in-Hospital Mortality by Endarterectomy Technique and Age Category (≤65 Years Versus >65 Years)

Variable	Analysis type	Studies (*n*)	Subgroups	Risk ratios (95% CI)	*P*-value	Clinical significance
Endarterectomy technique	Categorical	11	Open versus traction	Open: 1.56 (1.20-2.01); Traction: 1.45 (0.56-3.76)	.003	Significant difference between techniques
Age categories	Categorical	16	65 years versus >65 years	65 years: 1.90 (1.75-2.06); >65 years: 1.60 (1.24-2.07)	<.001	Younger patients show higher risk

Meta-regression analysis examined multiple study-level characteristics including sex distribution, left ventricular ejection fraction, diabetes prevalence, previous MI, hypertension, previous percutaneous coronary intervention, chronic obstructive pulmonary disease, 3-vessel disease prevalence, total bypass grafts, cardiopulmonary bypass time, and aortic cross-clamp time. None of these variables showed significant associations with treatment effects in univariable meta-regression analyses (**Table 4**).

**Table 4.** Results of Meta-Regression Analyses Examining the Association Between Study-Level Characteristics and Treatment Effects for 30-Day or in-Hospital Mortality

Variable	Studies (*n*)	Coefficient	*P*-value
Age	16	−0.0094	.805
Male percentage	16	0.0067	.673
LVEF	10	0.0438	.294
Diabetes (%)	15	0.0028	.52
Previous MI (%)	9	−0.0089	.353
Hypertension (%)	13	−0.0038	.185
Previous PCI (%)	6	0.0048	.775
COPD (%)	8	−0.0004	.974
Three-vessel disease (%)	7	0.0117	.682
Total bypass grafts	10	0.2953	.299
CPB time	10	−0.0029	.712
Aortic cross-clamp time	11	0.0014	.693

### Publication bias assessment

Assessment of publication bias for the primary outcome using Egger’s test showed no significant evidence of publication bias (*t* = −0.7531, *P* = .4639). The Doi plot demonstrated relatively symmetric distribution of studies (**Figure S2**), and LFK index was 0.132, indicating minor asymmetry and no substantial publication bias. Kendall’s tau correlation test similarly showed no significant publication bias (tau = 0.1000, *P* = .6259). Sensitivity analysis excluding the Kelly 2022 study showed consistent findings (LFK index 0.112).

## DISCUSSION

### Principal findings

This systematic review of 16 studies comprising 119 458 patients represents the most comprehensive synthesis of outcomes following CE combined with CABG. The analysis demonstrated increased perioperative morbidity and mortality in patients undergoing endarterectomy, with elevated rates of early death, MI, stroke, and renal failure compared with isolated CABG.

### Interpretation

These findings provide benchmarking data for 2 fundamentally different patient populations rather than evaluating a treatment effect. Patients requiring endarterectomy have complex coronary disease precluding standard grafting with few effective management strategies. The observed associations likely reflect underlying disease severity rather than harm from the technique itself.

This interpretation is supported by both individual and pooled adjusted analyses. When we pooled adjusted ORs from the 3 studies providing between-group estimates, the pooled adjusted OR was numerically lower than the crude pooled RR, and 2 of the 3 individual estimates (Tiruvoipati and Bagheri) did not reach statistical significance, suggesting that CE was not an independent predictor of mortality in these cohorts. Kelly et al found a statistically significant adjusted OR but also demonstrated that long-term hazards were time-dependent, with elevated risk only in the first year but not thereafter. More recently, Bagheri et al reported similar long-term mortality between CE-CABG and isolated CABG despite higher in-hospital mortality,^30^ further supporting that baseline disease severity drives much of the observed risk. These findings collectively indicate that the crude pooled estimate likely overestimates the independent effect of CE, with confounding by indication—whereby CE is applied to a sicker subpopulation—accounting for part of the observed excess. Nevertheless, the adjusted pooled estimate remains dominated by a single large study (Kelly 2022), and should therefore be interpreted as supportive rather than definitive.

The paradoxical finding that younger patients (≤65 years) demonstrated higher risk may reflect more aggressive disease in those developing severe coronary atherosclerosis prematurely, or surgical selection where younger patients with complex disease undergo more aggressive revascularization.

### Vessel-specific considerations

Across 6 studies reporting detailed vessel distributions (2961 endarterectomized vessels), the LAD artery (36.7%) and RCA (34.6%) were most frequently treated. Endarterectomy of the LAD artery was associated with increased perioperative MI risk,^26^ potentially reflecting the extensive myocardial territory at risk and technical challenges of complete plaque removal whilst preserving septal perforators. The choice of target vessel influences both procedural complexity and clinical outcomes.

Recent angiographic follow-up data reveal vessel-specific differences in graft patency. Tiemuerniyazi et al reported that whilst LAD endarterectomy achieved 80% graft patency, RCA endarterectomy demonstrated significantly lower patency and was independently associated with increased graft failure risk despite comparable surgical technique and pharmacotherapy protocols.^31^

### Technical variations

Endarterectomy techniques varied from limited traction methods to extensive open endarterectomy with patch reconstruction. Open techniques allow complete visualization but require longer arteriotomies, whilst traction methods are technically simpler but risk incomplete plaque removal. Subgroup analysis revealed significant differences between techniques, with open endarterectomy showing higher risk ratios compared with traction endarterectomy. The choice between primary closure and patch reconstruction remains surgeon-dependent, with patch use potentially reducing anastomotic stenosis in smaller vessels. These technical variations contributed to outcome heterogeneity across studies.

### Postoperative pharmacological management

The extensive endothelial injury following CE creates a prothrombotic environment, yet our review reveals considerable heterogeneity in postoperative antiplatelet and anticoagulation protocols, with no standardized guidelines to inform practice.

Dual antiplatelet therapy (DAPT) was the predominant approach. Abid et al administered aspirin 75 mg with clopidogrel 75 mg daily for 3 months, alongside unfractionated heparin, arguing that 2 agents provide superior protection when thrombotic risk is highest.^23^ LaPar et al similarly maintained DAPT for at least 3 months but avoided routine anticoagulation.^22^ Chi et al extended DAPT to 1 year before transitioning to lifelong aspirin.^20^ Contemporary evidence from Balaj et al demonstrated that patients receiving DAPT for 6 months achieved significantly higher graft patency and lower rates of mortality and major adverse events compared with aspirin alone during a mean follow-up of 97 months.^32^

The role of anticoagulation remains contentious. Ferraris et al argued that the post-endarterectomy milieu resembles that after percutaneous intervention, where aspirin alone proves insufficient.^28^ They routinely administered warfarin for 2–3 months, accepting the bleeding risks for enhanced antithrombin activity. Erdil et al employed similar 6-month anticoagulation protocols.^27^ Emerging evidence suggests warfarin may confer superior graft patency. In a recent retrospective cohort study, Zhen et al compared warfarin plus aspirin for 3 months (followed by DAPT) against DAPT alone. The warfarin group demonstrated significantly higher graft patency and longer median time to graft failure without increased bleeding complications. These findings suggest short-term anticoagulation may improve outcomes, though prospective validation is needed.^33^

However, several contemporary series achieved acceptable outcomes without anticoagulation. Ali et al documented striking variation even within a single institution: 43.8% of patients received direct oral anticoagulants for 3 months, 27.1% received warfarin, and 29.2% received only DAPT.^34^ One patient in da Costa’s series who developed stroke after discharge was not receiving clopidogrel, suggesting potential consequences of inadequate coverage.^16^

Current practice favours DAPT for 3–12 months, with lifelong aspirin thereafter. Whilst anticoagulation use has historically been driven by institutional preference, emerging retrospective evidence suggests warfarin may improve graft patency. Two ongoing randomized controlled trials—PATH-CARE (comparing DAPT versus DAPT plus warfarin) and THACE-CABG (comparing tirofiban versus heparin for bridging)—will provide more definitive evidence to guide optimal antithrombotic strategies following CE.^35^^,^^36^

### Clinical implications

Despite increased perioperative risk, CE enables complete revascularization in patients with diffuse disease who would otherwise be inoperable or face incomplete revascularization. Patient counselling must acknowledge short-term risk whilst emphasizing that endarterectomy often provides the only means of surgical revascularization.

The findings support the importance of careful patient selection and discussion about realistic outcomes. Contemporary risk prediction tools incorporating age, left main disease, mitral regurgitation, and LAD endarterectomy can estimate 1- and 3-year risk of major adverse events, facilitating counselling.^37^ Novel biomarkers may further improve risk stratification. Preoperative D-dimer levels ≥0.235 μg/mL independently predict perioperative major adverse events,^38^ whilst coronary plaque burden exceeding 1.15 cm is associated with 5-fold increased risk of perioperative MI.^39^

Surgeon experience and institutional volume likely influence results, though our analysis could not directly assess these factors. Centres performing CE should maintain sufficient volume to ensure technical proficiency, particularly for complex procedures.

### Limitations

This study has several important limitations. Most fundamentally, patients requiring CE represent a distinct disease phenotype with diffuse, calcified, end-stage coronary disease that precludes conventional grafting. The comparison group has focal lesions amenable to standard bypass. This inherent selection bias cannot be eliminated through statistical adjustment, as the decision to perform endarterectomy reflects disease severity not fully captured by measured covariates. Our findings therefore provide benchmarking data for counselling patients when endarterectomy is necessary, rather than suggesting the technique causes harm. For many of these patients, the alternative is incomplete revascularization or medical management alone.

Second, most included studies are observational, and confounding by indication is an inherent concern. Although we attempted to address this by pooled adjusted estimates, only 3 studies provided between-group adjusted ORs for operative mortality, and the pooled adjusted analysis is dominated by a single large study. The adjusted pooled result should therefore be interpreted as supportive rather than definitive. Important variables such as extent of CAD, number of grafts, arterial grafting, and urgency of surgery remain underreported across the evidence base, further limiting the precision of confounder adjustment.

Third, substantial clinical and methodological heterogeneity exists across studies, including differences in CE technique (traction versus open endarterectomy; with or without patch reconstruction), anticoagulation and antiplatelet protocols, outcome definitions, and follow-up periods. CE technique was reported in only 50% of included studies, and vessel distribution data in only 37.5%, precluding definitive subgroup analyses by technique or target vessel. Nevertheless, the consistency of findings across all sensitivity analyses provides reassurance that the overall result is robust.

Fourth, whilst our inclusion criteria specified isolated CABG procedures, we cannot completely exclude the possibility that some patients in the included studies underwent minor concomitant procedures (such as left atrial appendage occlusion or pulmonary vein isolation) where procedural details may not be fully captured. However, 14 of 16 studies explicitly confirmed isolated CABG populations, and sensitivity analysis restricted to these 14 studies yielded a virtually identical estimate, suggesting that any such contamination has minimal impact on the overall result.

Fifth, one large database study (Kelly 2022) contributed substantial weight to several analyses, potentially influencing overall results. Sensitivity analyses excluding this study showed a consistent direction of effect with a somewhat attenuated point estimate, confirming that findings are not solely driven by this single study. Finally, limited availability of long-term outcome data, long-term angiographic follow-up, and detailed surgical data prevented comprehensive assessment of graft durability and late clinical outcomes, and reduced the clinical granularity of this analysis.

## CONCLUSION

CE combined with CABG is associated with higher early mortality and morbidity compared with isolated CABG. However, confounder-adjusted analyses suggest that the excess risk is at least partially attributable to the greater baseline disease severity inherent to the CE population, rather than being entirely an independent effect of the endarterectomy technique. The scarcity of adjusted data, the absence of randomized evidence, and incomplete reporting of key surgical variables preclude definitive causal conclusions.

These findings provide benchmarking data for counselling patients about expected outcomes when endarterectomy is necessary to achieve complete revascularization. Future studies with standardized reporting of CE technique, vessel distribution, and confounder-adjusted comparisons are needed to better define the independent contribution of CE to perioperative risk. Two ongoing randomized controlled trials (PATH-CARE and THACE-CABG) will provide higher-quality evidence to inform clinical practice.

## Supplementary Material

ivag091_Supplementary_Data

## Data Availability

The data underlying this article are publicly available due to the nature of systematic reviews.
