# Supplementary material for: Short-Term Outcomes of Coronary Endarterectomy as an Adjunct to Coronary Artery Bypass Grafting: A Systematic Review and Meta-Analysis of Over 100 000 Patients
Source: Interdiscip Cardiovasc Thorac Surg. 2026 Mar 25;41(4):ivag091. doi: 10.1093/icvts/ivag091 (PMC13070702; doi:10.1093/icvts/ivag091)
Supplement: ivag091_Supplementary_Data [file ivag091_supplementary_data.zip › R2.Supplementary_Materials.docx]

# Supplementary Figures/Tables

**Supplementary Figure 1:** Funnel plot for assessment of publication bias in studies reporting 30-day or in-hospital mortality.


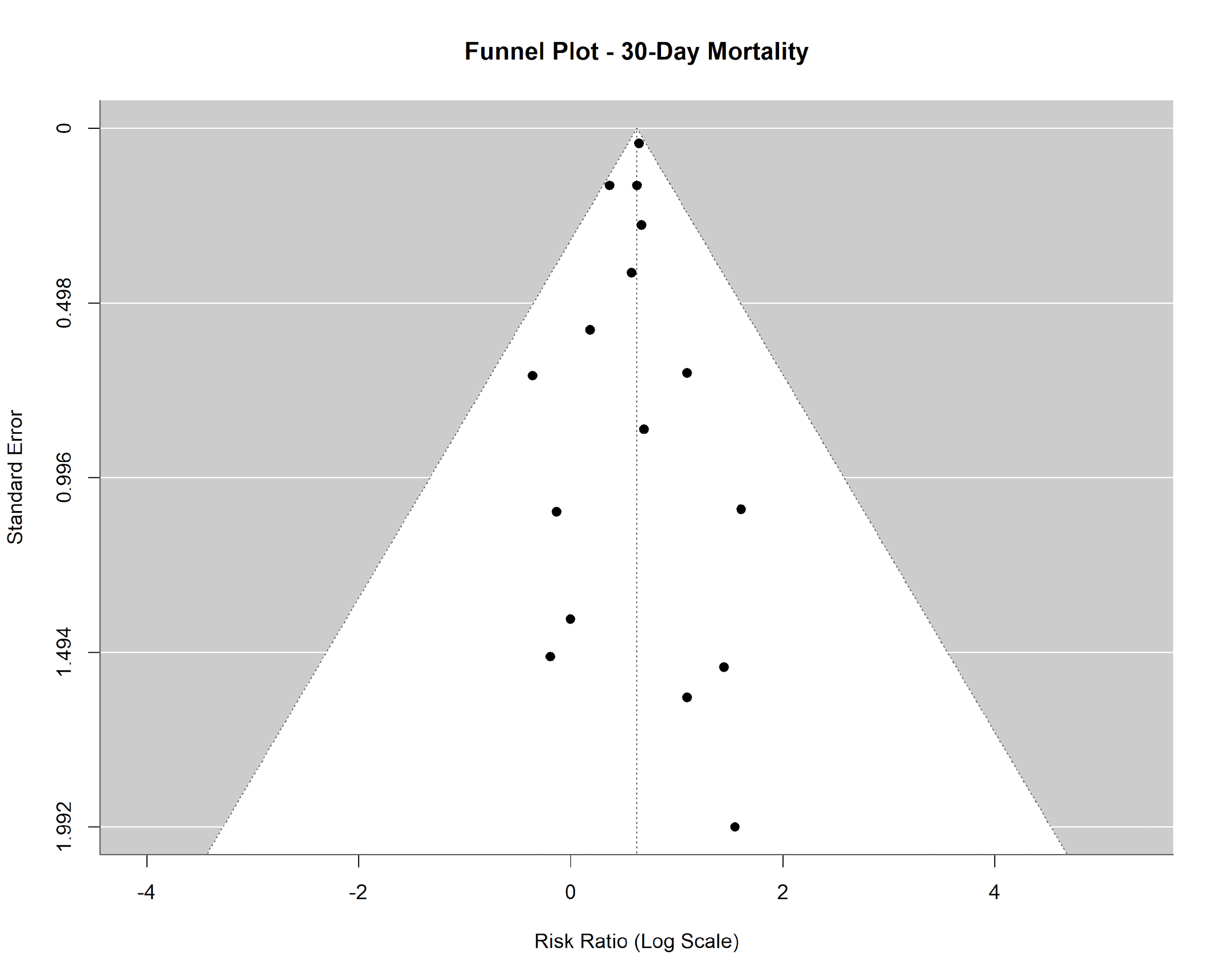


**Supplementary Figure 2.** Doi plots for assessment of publication bias in studies reporting 30-day or in-hospital mortality. (A) All studies. (B) Sensitivity analysis excluding Kelly 2022.

**
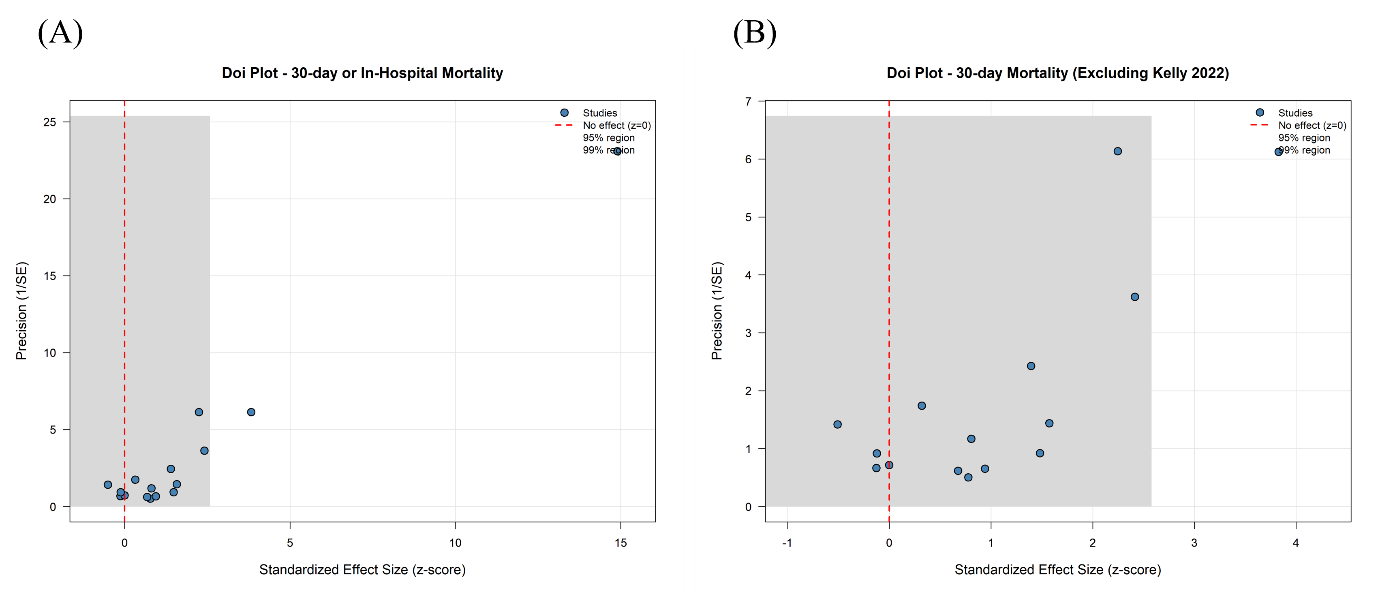
**

**Supplementary Table 1:** Study quality assessment using the Newcastle-Ottawa Scale.

| **Author** | **Year** | **Selection** |  |  |  | **Comparability** |  | **Outcome** |  |  | **Total** | **Quality** |
| --- | --- | --- | --- | --- | --- | --- | --- | --- | --- | --- | --- | --- |
|  |  | Representativeness | Selection | Exposure | Outcome | Controls | Additional controls | Assessment | Follow-up | Adequacy | Stars | Rating |
| Kelly | 2022 | * | * | * | * | * | * | * | * | * | 9 | Good |
| Eris | 2021 | * | * | * | * | * | - | * | * | * | 8 | Fair |
| Sabzi | 2020 | * | * | * | * | * | - | * | * | * | 8 | Fair |
| CostaMACD | 2020 | * | * | * | * | * | - | * | * | * | 8 | Fair |
| Janiec | 2019 | * | * | * | * | * | * | * | * | * | 9 | Good |
| Toker | 2017 | * | * | * | * | * | - | * | * | * | 8 | Fair |
| Bagheri | 2016 | * | * | * | * | * | - | * | * | * | 8 | Fair |
| Chi | 2015 | * | * | * | * | * | - | * | * | * | 8 | Fair |
| Binsalamah | 2014 | * | * | * | * | * | * | * | * | * | 9 | Good |
| LaPar | 2011 | * | * | * | * | * | - | * | * | * | 8 | Fair |
| Abid | 2009 | * | * | * | * | * | - | * | * | - | 7 | Fair |
| Sirivella | 2005 | * | * | * | * | * | * | * | * | * | 9 | Good |
| Tiruvoipati | 2005 | * | * | * | * | * | - | * | * | * | 8 | Fair |
| Silberman | 2002 | * | * | * | * | * | - | * | * | * | 8 | Fair |
| Erdil | 2002 | * | * | * | * | * | - | * | * | - | 7 | Fair |
| Khilji | 2020 | * | * | * | - | - | - | * | - | - | 4 | Poor |

**Supplementary Table 2.** Reporting completeness across included studies

| **Variable** | **Studies reporting (n)** | **Percentage (%)** |
| --- | --- | --- |
| 30-day/in-hospital mortality (primary) | 16 | 100.0 |
| Perioperative MI | 14 | 87.5 |
| Stroke | 9 | 56.3 |
| Renal failure | 7 | 43.8 |
| Reoperation for bleeding | 5 | 31.3 |
| ICU length of stay | 9 | 56.3 |
| IABP use | 8 | 50.0 |
| Postoperative AF | 8 | 50.0 |
| Long-term mortality | 10 | 62.5 |
| CE technique details | 8 | 50.0 |
| Vessel distribution | 6 | 37.5 |
| Between-group adjusted effect estimates | 3 | 18.8 |
| Explicitly isolated CABG confirmed | 14 | 87.5 |
| MI = myocardial infarction; ICU = intensive care unit; IABP = intra-aortic balloon pump; AF = atrial fibrillation; CE = coronary endarterectomy; CABG = coronary artery bypass grafting. Rows above the dashed line are clinical outcomes; rows below are study-level characteristics. | | |
