# Supplementary figures and images for: Short-Term Outcomes of Coronary Endarterectomy as an Adjunct to Coronary Artery Bypass Grafting: A Systematic Review and Meta-Analysis of Over 100 000 Patients
Source: Interdiscip Cardiovasc Thorac Surg. 2026 Mar 25;41(4):ivag091. doi: 10.1093/icvts/ivag091 (PMC13070702; doi:10.1093/icvts/ivag091)

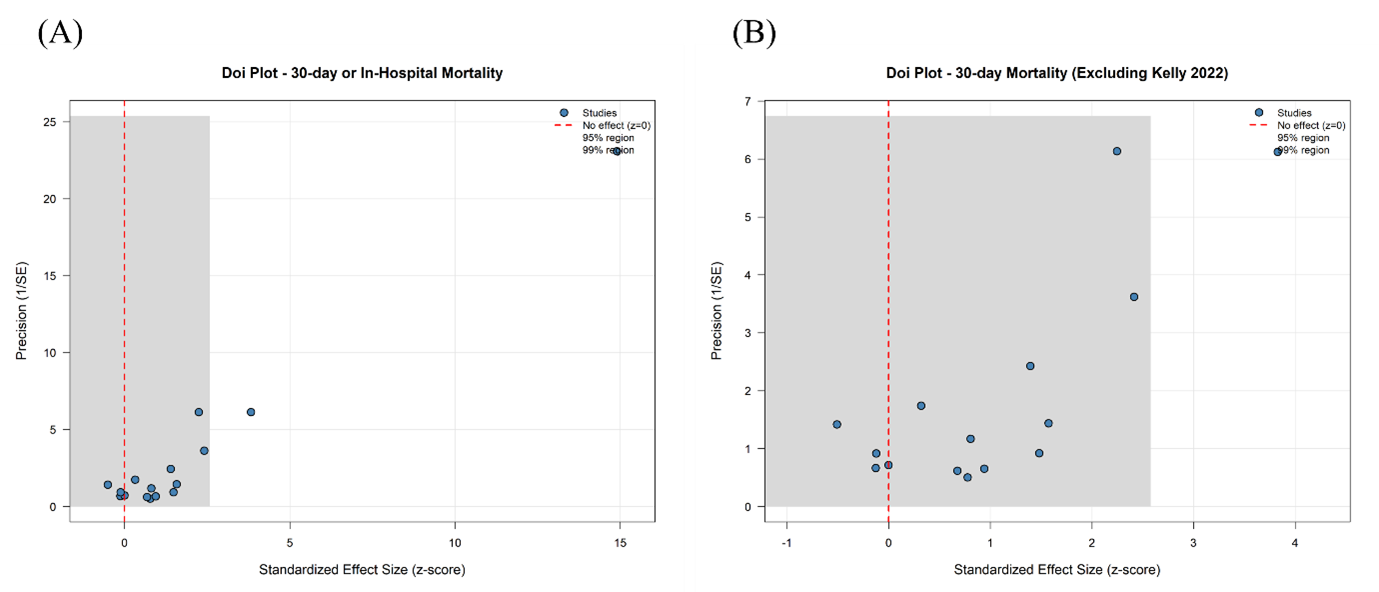

Supplement: ivag091_Supplementary_Data [file ivag091_supplementary_data.zip › R2.Supplementary_Figure_2.tif]

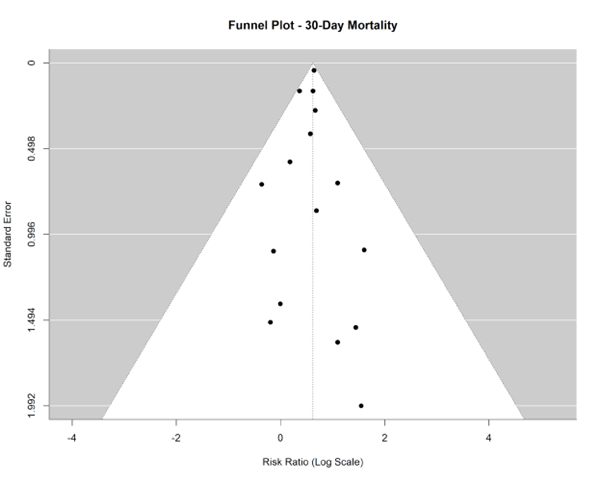

Supplement: ivag091_Supplementary_Data [file ivag091_supplementary_data.zip › R2.Supplementary_Figure_1.tif]
